# Integrated Analysis of Expression and Prognostic Values of Acyl-CoA Dehydrogenase short-chain in Colorectal Cancer

**DOI:** 10.7150/ijms.63953

**Published:** 2021-09-07

**Authors:** Qi Wu, Tao Yan, Yijiao Chen, Jiang Chang, Yudong Jiang, Dexiang Zhu, Ye Wei

**Affiliations:** 1 Department of General Surgery, Zhongshan Hospital, Fudan University, Shanghai, 200032, China.; 2 Department of Cardiovascular Surgery, Zhongshan Hospital, Fudan University, Shanghai, 200032, China.

**Keywords:** ACADS, bioinformatics, colorectal cancer, fatty acid metabolism, immune infiltration

## Abstract

**Background:** Acyl-CoA dehydrogenase short-chain (ACADS) is a crucial enzyme in the fatty acid metabolism pathway located in mitochondria. However, the expression level and prognostic value of ACADS in colorectal cancer (CRC) remain unclear.

**Methods:** The mRNA and protein expression data of ACADS was obtained from The Cancer Genome Atlas (TCGA), Clinical Proteomic Tumor Analysis Consortium (CPTAC), and Oncomine. Prognostic values of ACADS were calculated using Kaplan-Meier survival analysis. Correlations between ACADS and immune infiltration were estimated using TIMER, CIBERSORT, EPIC, quanTIseq, and xCell. The UALCAN and MEXPRESS databases were utilized for Methylation analysis. The co-expression analysis based on mRNA expression and interaction network of ACADS were performed via several online tools. Gene Ontology (GO) and Kyoto Encyclopedia of Genes and Genomes (KEGG) analysis on ACADS co-expressed genes were performed using the Metascape.

**Results:** The expression analysis demonstrated that ACADS was down-regulated in CRC tissues compared with paired normal tissue. Expression of ACADS was found to be significantly associated with clinical cancer stages and the consensus molecular subgroups (CMS) constituent ratio in CRC patients. Besides, lower ACADS expression was found to predict poor prognosis and be significantly associated with common immune checkpoint genes and MMR genes in CRC. ACADS expression levels were positively related to B cells, CD4^+^ T cells, CD8^+^ T cells, M1 macrophages, neutrophils, and Tregs, while negatively correlated with M0 macrophages, M2 macrophages. The methylation level of ACADS in normal tissues was significantly higher than that in tumor tissues, and several methylation sites were identified. The enrichment analysis suggested the co-expressed genes mainly enriched in cell mitochondrial metabolism.

**Conclusions:** The present study provided multilevel evidences for expression of ACADS in CRC and the function of ACADS in prognostic prediction, immune infiltration, and methylation. ACADS might have the potential as the novel biomarker and therapeutic target in CRC patients.

## Introduction

Colorectal cancer (CRC) is the second most common reason for cancer death in the world, and the most commonly diagnosed malignancy 1. The 5-year survival rate ranges from higher than 90% in CRC patients with localized disease to about 14% in patients with the distant-stage disease 2. Most advanced CRC patients have a poor prognosis because of distant metastasis 3. Alterations in genetic levels of oncogenes or tumor suppressors play a vital role in CRC. Despite the fact that some excellent progress has been made, work still has to be done to uncover the specific mechanism in CRC 4. A better understanding of the biological mechanisms in CRC development is essential for finding new biomarkers and therapeutic targets.

Cancer metabolism is an essential symbol of tumorigenesis and development and is also vital in the development of anticancer drugs. Multiple altered metabolic pathways lead to faster cancer growth and metastasis 5. Lipids are orchestrated by a series of highly complex molecules, which not only take part in the construction of biofilms but also have the ability to transmit signals. The enhanced and changed lipid synthesis facilitates the rapid growth of tumor cells, and targeting lipid metabolism may be a potential strategy for cancer treatment 6. Most of the lipids are transferred from fatty acids (FAs), and enhanced synthesis of FA in various cancers is confirmed and changed FA synthesis was demonstrated to contribute to tumorigenesis in many cancers 7. Fatty acid oxidation (FAO) or fatty acid beta-oxidation is an important process to generate Adenosine Triphosphate (ATP) and was associated with cancer cell growth and survival in various cancers 8. Recent studies indicated that inhibition of critical enzymes in FAO showed tumor-suppressive activity in breast cancer and malignant glioma cells 9,10.

Previous studies showed that different expression genes associated with fatty acid metabolic pathways might take part in the development and metastasis of CRC 11,12. The enzyme acyl-CoA dehydrogenase short-chain (ACADS) catalyzes the first step of the mitochondrial fatty acid beta-oxidation, which is supposed to have a role in the carcinogenesis of CRC. However, the specific function of ACADS and the related regulatory networks are still unknown in CRC.

In the present study, we analyzed data from diverse public databases utilizing various bioinformatics tools, in order to explore the expression and influence of ACADS in CRC, and further investigated the potential mechanism of ACADS in CRC. We hope this study can provide potential therapeutic targets and new research ideas for treatment of CRC.

## Methods

### Data acquisition

Four independent public datasets, GSE17536, GSE17538, GSE24551 and GSE39582 were downloaded from the Gene Expression Omnibus (GEO) database for further analysis. RNA sequencing data and clinical data of CRC patients were acquired from The Cancer Genome Atlas (TCGA). Proteomics data of CRC was downloaded from Clinical Proteomic Tumor Analysis Consortium (CPTAC). Seven datasets of CRC, including Notterman Colon, Skrzypczak Colorectal, Skrzypczak Colorectal 2, Kaiser Colon, Hong Colorectal, Sabates-Beliver Colon, and Ki Colon, were obtained from the Oncomine database. Different tumor cell lines data were acquiered from Cancer Cell Line Encyclopedia (CCLE) database 13. Immunohistochemistry (IHC) staining images were collected from the Human Protein Atlas (HPA) 14.

### Patient selection

A total of twenty patients pathologically diagnosed with CRC were included in this study. Tumor tissues and paired adjacent tissues were collected during surgery, then immediately preserved in liquid nitrogen and transferred to a refrigerator at -80 °C for further study. This study was in full compliance with the Declaration of Helsinki and approved by the Medical Ethics Committee of Zhongshan Hospital, Fudan University. Written informed consent was obtained from all patients participating in this study before surgery.

### Quantitative real-time PCR (qRT-PCR)

Total cellular RNA from tumor tissues and paired adjacent tissues were extracted following the manufacturer's instruction. The Complementary DNA (cDNA) was synthesized by reverse transcription at 42 °C for 60 minutes and then at 95 °C for 5 minutes. QRT-PCR was performed at the temperature of 95 °C for 30 seconds, followed by 40 cycles with the temperature of 95 °C for 5 seconds and 60 °C for 34 seconds. The expression of RNA levels was normalized by GAPDH, and the 2^-ΔΔCT^ method was applied with three independent repeats. All sequences for RNA primers (Sangon Biotech, Shanghai, China) are shown in Table 1.

### Survival analysis

The associations between the overall survival (OS), disease-free survival (DFS), and relapse-free survival (RFS) of CRC patients and expression levels of ACADS were calculated using Kaplan-Meier survival analysis. Univariate Cox analysis and Log-rank test were performed to determine hazard ratio (HR) with 95% confidence intervals and *p*-value, respectively.

### Construction of the miRNA-mRNA network

The prediction of target miRNA is vital for identifying the function and mechanism of ACADS. The online tool, Starbase, which included seven online databases for potential miRNA-mRNA interaction, was used for prediction to ensure the integrity of target miRNAs.

### Immune infiltration

Immune infiltration plays vital roles in CRC. Different algorithms, including TIMER 15, CIBERSORT 16, EPIC 17, quanTIseq 18, and xCell 19, were utilized to estimate correlations between immune infiltration and ACADS.

### Methylation analysis

DNA methylation is a chemical modification, which causes changes in chromatin structure, DNA conformation, DNA stability and the interaction between DNA and protein, thus controlling gene expression. The UALCAN 20, was applied to analyze methylation levels of ACADS between CRC and normal tissues. DNA sequences and the methylation sites of DEGs were explored utilizing MEXPRESS 21.

### Co-expression analysis

The LinkedOmics database 22, a website tool based on 32 TCGA cancer-associated multi-dimensional datasets, was used to conduct the co-expression analysis of ACADS using Pearson's correlation coefficient. The top 100 positively correlated co-expressed genes were imported into the Search Tool for the Retrieval of Interacting Genes (STRING) 23 to generate the protein-protein interaction (PPI) network. Nodes represent proteins, and edges represent protein-protein associations in the PPI network. The results downloaded from the STRING database was then visualized utilizing Cytoscape software. Metascape 24, a webtool for gene function annotation, was applied to perform enrichment analysis.

## Results

### Expression levels of ACADS

**Figure 1:**
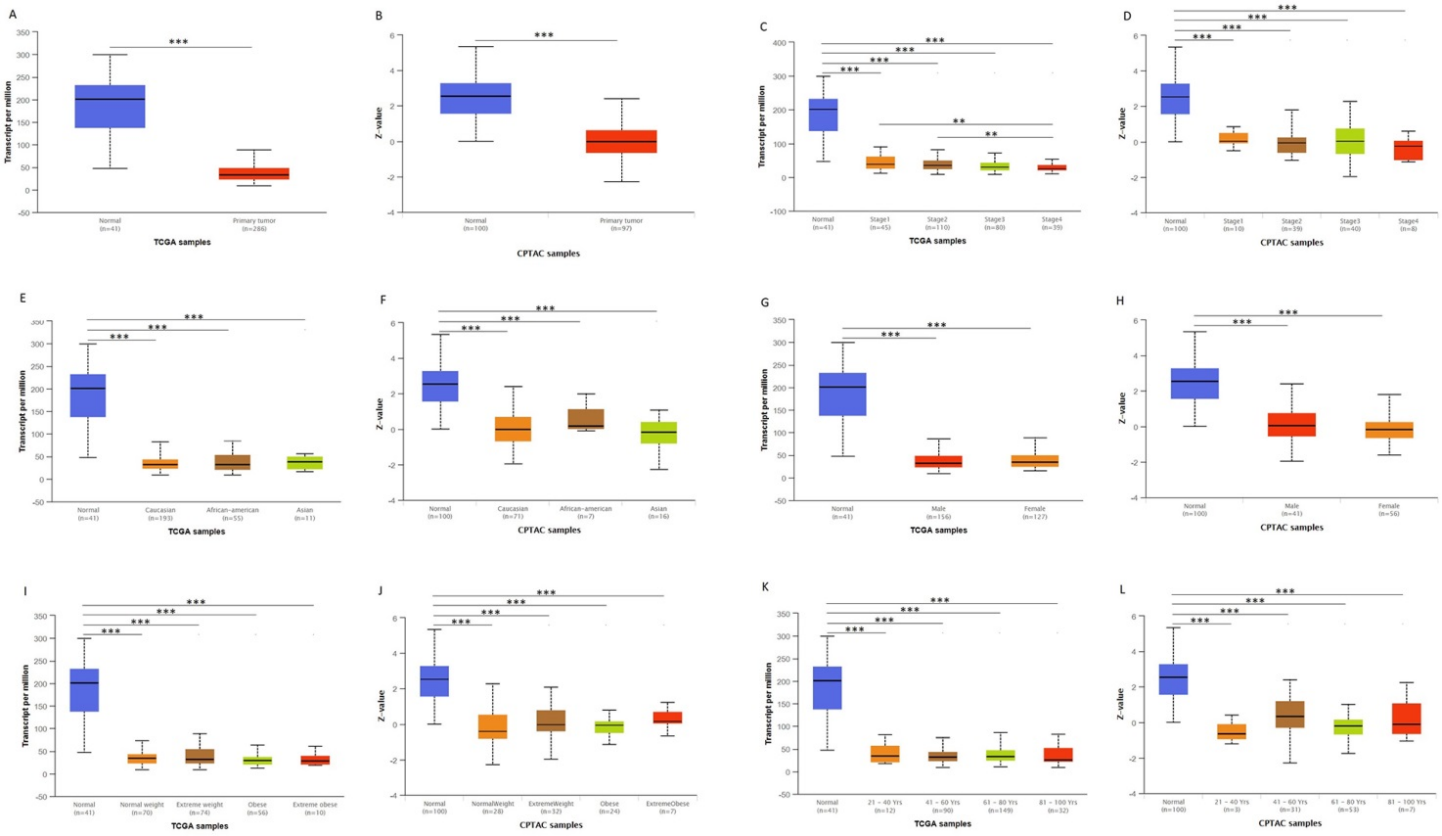
** ACADS mRNA and protein levels in subgroups of patients with CRC from TCGA and CPTAC. (A and B)** Relative expression of ACADS in normal and CRC samples. **(C and D)** Relative expression of ACADS in normal individuals and in CRC patients with different stages. **(E and F)** Relative expression of ACADS in different races. **(G and H)** elative expression of ACADS in different genders. **(I and J)** Relative expression of ACADS in patients with different weights. **(K and L)** Relative expression of ACADS in different ages. *p < 0.05, **p < 0.01, ***p < 0.001.

**Figure 2:**
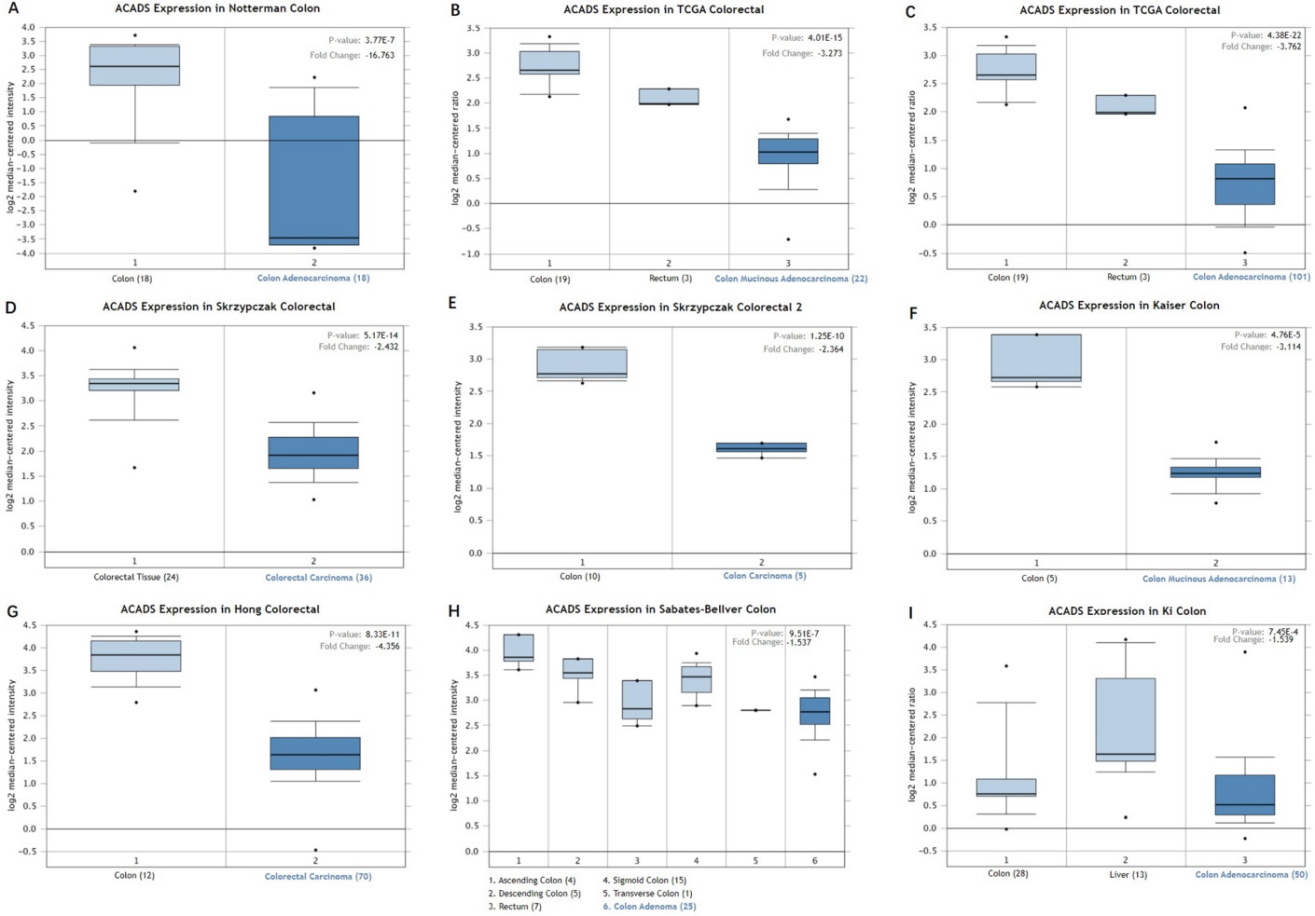
** Expression levels of ACADS between CRC tissues and normal tissues. (A)** Colon adenocarcinoma vs. Colon. **(B)** Colon mucinous adenocarcinoma vs. Colon and Rectum. **(C)** Colon adenocarcinoma vs. Colon and Rectum. **(D)** Colorectal carcinoma vs. Colorectal tissue. **(E)** Colon carcinoma vs. Colon. **(F)** Colon mucinous adenocarcinoma vs. Colon. **(G)** Colorectal carcinoma vs. Colon. **(H)** Colon adenoma vs. Ascending colon, Descending colon, Rectum, Sigmoid colon and Transverse colon. **(I)** Colon adenocarcinoma vs. Colon and Liver.

We first analyzed the data of each tumor cell line downloaded from the CCLE database, and examined the expression level of ACADS in diverse tumor tissues according to the tissue source. As shown in Figure S1, expression levels of ACADS in 63 CRC cell lines were maintained at 2-4, which was relatively higher than other cancer cell lines. Compared with paired normal tissues, expression levels of ACADS were significantly downregulated in tumor tissues (Figure S2). We also performed qRT-PCR using tumor tissues and paired adjacent tissues from CRC patients in our hospital. The result was consistent with public database (Figure S3). Furthermore, we verified ACADS expression levels both in mRNA and protein levels in different subtypes of CRC in TCGA and CPTAC. The results demonstrated that ACADS expression diminished both in mRNA and protein levels, and was associated with tumor stage, race, gender, weight, and age (Figure 1). Seven independent datasets containing tumor tissues and normal tissues expression profile were selected for analysis to further verify expression levels of ACADS (Figure 2). It revealed that ACADS expression in tumor tissues was generally lower than that in normal colon tissues. IHC results demonstrated that the staining levels of ACADS were low in CRC tissues while high in normal tissues (Figure S4).

### Associations between ACADS and clinical characteristics

To better understand the potential function of ACADS, we divided CRC patients into two groups, including ACADS-high group, and ACADS-low group, using the cut-off value calculated by the X-tile program. TCGA results suggested that the ACADS-low group had advanced N stage, M stage, Pathologic stage, and lymph nodes ratio (NLR). In addition, microsatellite instability (MSI) state and consensus molecular subgroups (CMS) were different significantly in two groups (Table S1). Similarly, it demonstrated that the ACADS-low group had higher III-IV stage proportion, and the CMS constituent ratio was different between two groups as well (Table S2). In addition, ACADS expression levels were positively related to deficiency in DNA mismatch repair enzymes (dMMP). In addition, we found that ACADS was negatively associated with mismatch repair (MMR) gene PMS2 and positively associated with immune checkpoint gene TNFRSF14, LGALS9, and TMIGD2 in TCGA (Figure S5).

### Prognostic values of ACADS

**Figure 3:**
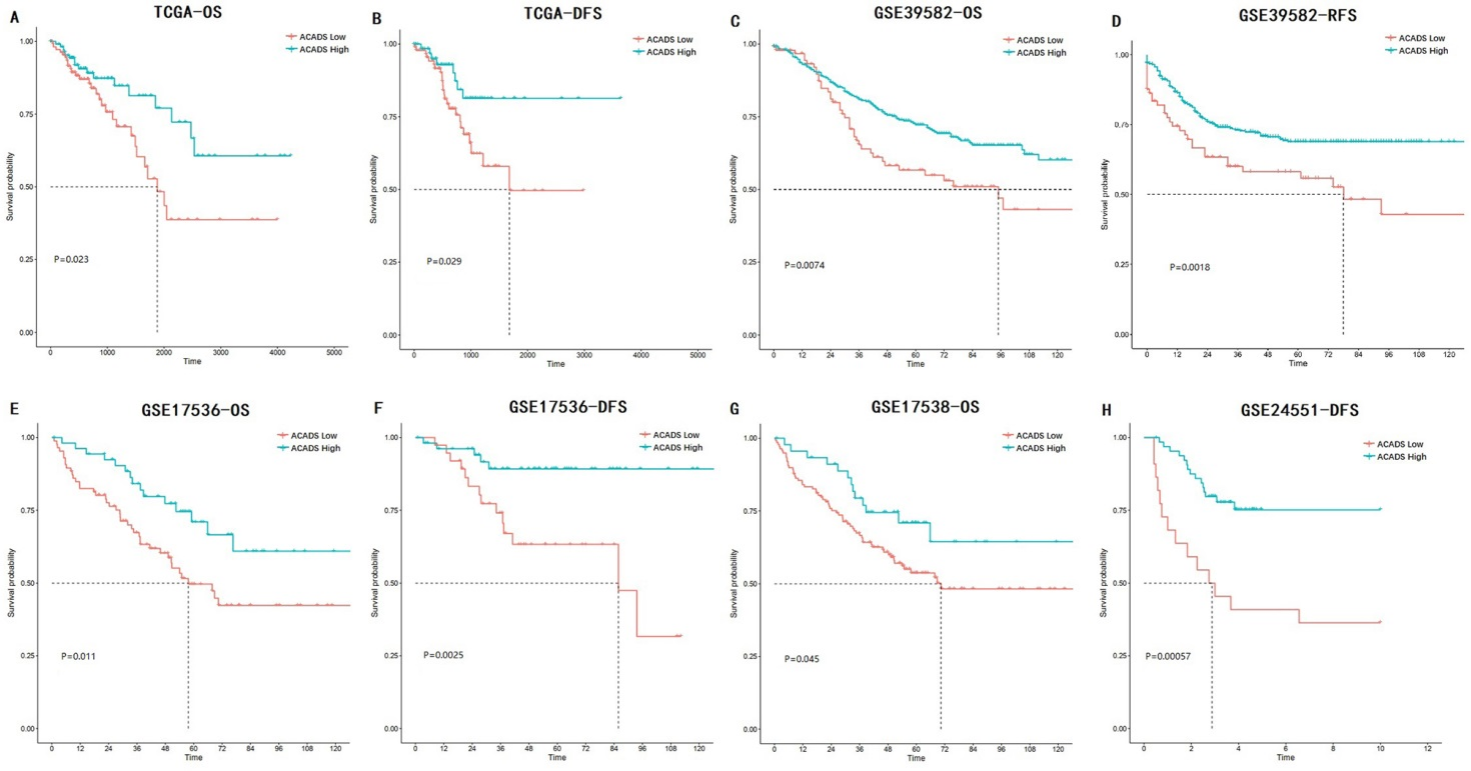
** Survival analysis of ACADS in colorectal cancer (CRC). (A)** The overall survival (OS) of CRC patients in TCGA. **(B)** The disease-free survival (DFS) of CRC patients in TCGA. **(C)** The OS of CRC patients in GSE39582. **(D)** The relapse-free survival (RFS) of CRC patients in GSE39582. **(E)** The OS of CRC patients in GSE17536. **(F)** The DFS of CRC patients in GSE17536. **(G)** The OS of CRC patients in GSE17538. **(H)** The DFS of CRC patients in GSE24551.

The associations between *ACADS* expression levels and prognosis in various CRC datasets were analyzed using univariate Cox analysis and Kaplan-Meier survival analysis. Increased expression levels of ACADS were significantly associated with longer OS rate in TCGA, GSE39582, GSE17536, and GSE17538. We also calculated correlations between ACADS and DFS, which was consistent with OS analysis (Figure 3).

### Construction of the miRNA-mRNA network

**Figure 4:**
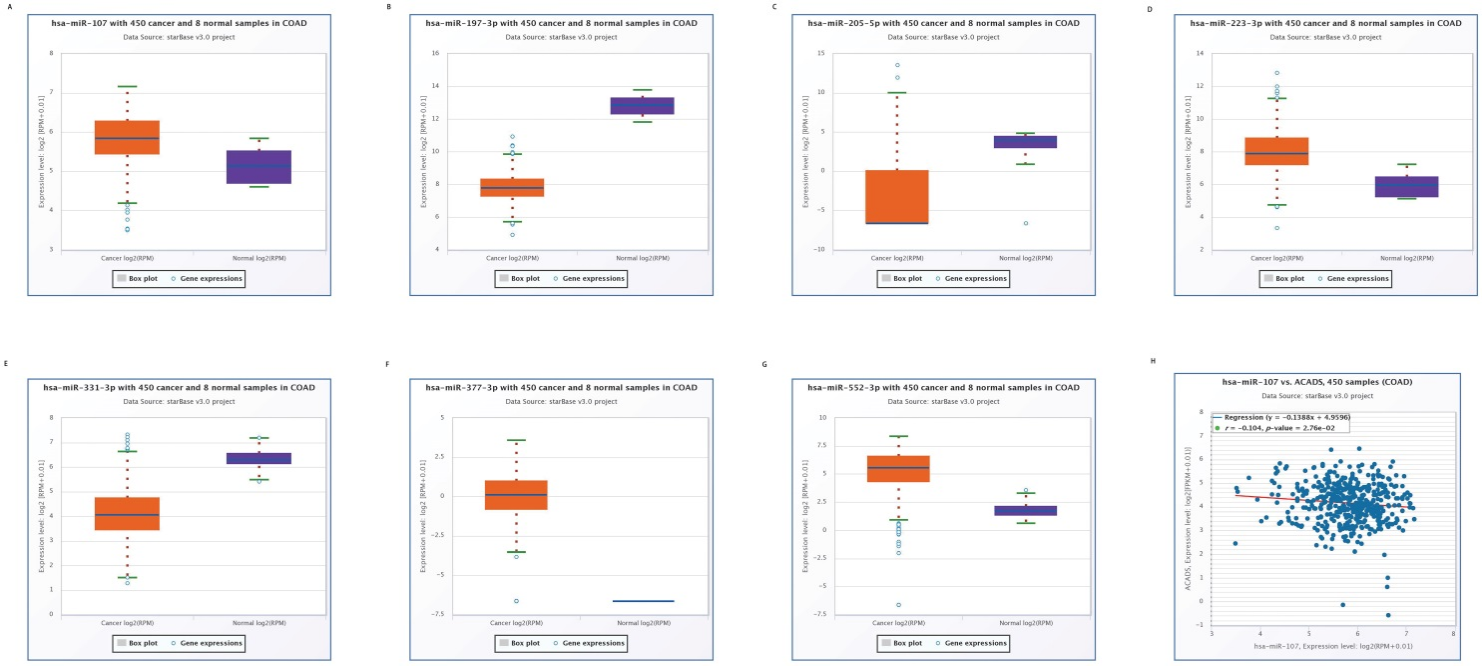
** Expression levels of predicted miRNAs between CRC tissues and normal tissues. (A)** Expression levels of miR-107. **(B)** Expression levels of miR-197-3p. **(C)** Expression levels of miR-205-5p. **(D)** Expression levels of miR-223-3p. **(E)** Expression levels of miR-331-3p. **(F)** Expression levels of miR-377-3p. **(G)** Expression levels of miR-552-3p. **(H)** Correlations between miR-107 and ACADS.

We used the Starbase online tool to predict upstream miRNAs which could potentially regulate ACADS, and a total of 11 miRNAs were identified. Moreover, we calculated expression levels of 11 miRNAs between tumor tissues and normal tissues. Seven miRNAs, including miR-552-3p, miR-331-3p, miR-377-3p, miR-223-3p, miR-205-5p, miR-197-3p, and miR-107 were identified as different between cancer and para-cancerous tissues. Considering the regulatory relationship between miRNA and mRNA is generally negative, we further analyzed the correlations between these seven miRNAs and ACADS to ensure that predicted miRNAs were potential upstream targets of ACADS. The result demonstrated that miR-107 was negatively correlated with expression levels of ACADS (Figure 4). Targeted therapy for miR-107 may increase the expression of ACADS to achieve a better prognosis.

### Immune infiltration

**Figure 5:**
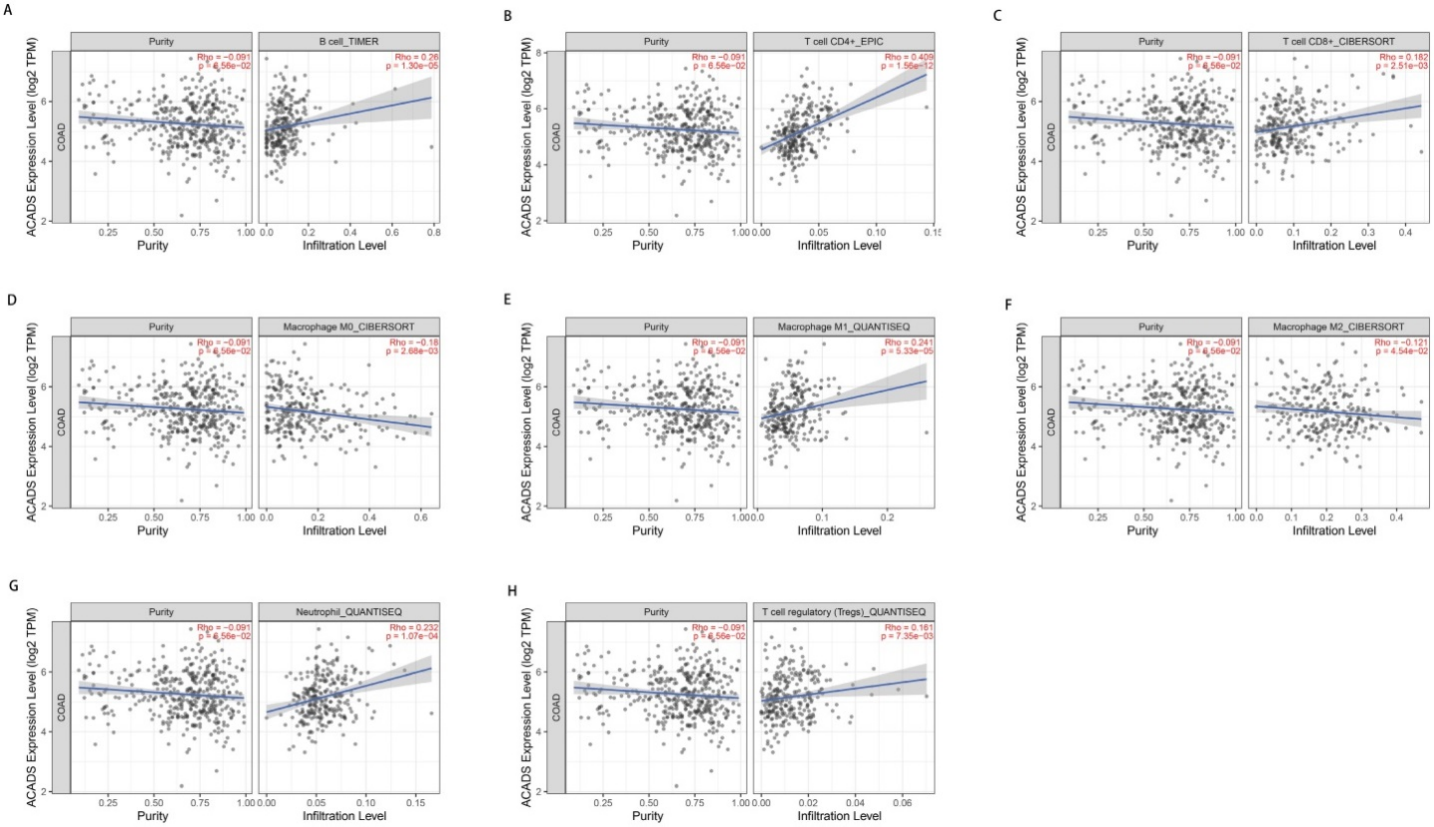
** Correlations between ACADS and immune infiltration. (A)** Correlations between ACADS and B cells. **(B)** Correlations between ACADS and CD4^+^ T cells. **(C)** Correlations between ACADS and CD8^+^ T cells. **(D)** Correlations between ACADS and M0 macrophages. **(E)** Correlations between ACADS and M1 macrophages. **(F)** Correlations between ACADS and M2 macrophages. **(G)** Correlations between ACADS and neutrophils. **(H)** Correlations between ACADS and Tregs.

In consideration of the crucial role of immune infiltration in the pathophysiology of CRC, diverse algorithms were utilized after tumor purity adjustment to analyze correlations between ACADS gene expression levels and immune infiltration. We found that ACADS expression levels were positively related to B cells (r = 0.26, p < 0.01), CD4^+^ T cells (r = 0.409, p < 0.01), CD8+ T cells (r = 0.182, p < 0.01), M1 macrophages (r = 0.241, p < 0.01), neutrophils (r = 0.232, p < 0.01), and Tregs (r = 0.161, p < 0.01), while negatively correlated with M0 macrophages (r = -0.18, p < 0.01), M2 macrophages (r = -0.121, p < 0.05, Figure 5).

### Methylation analysis

**Figure 6:**
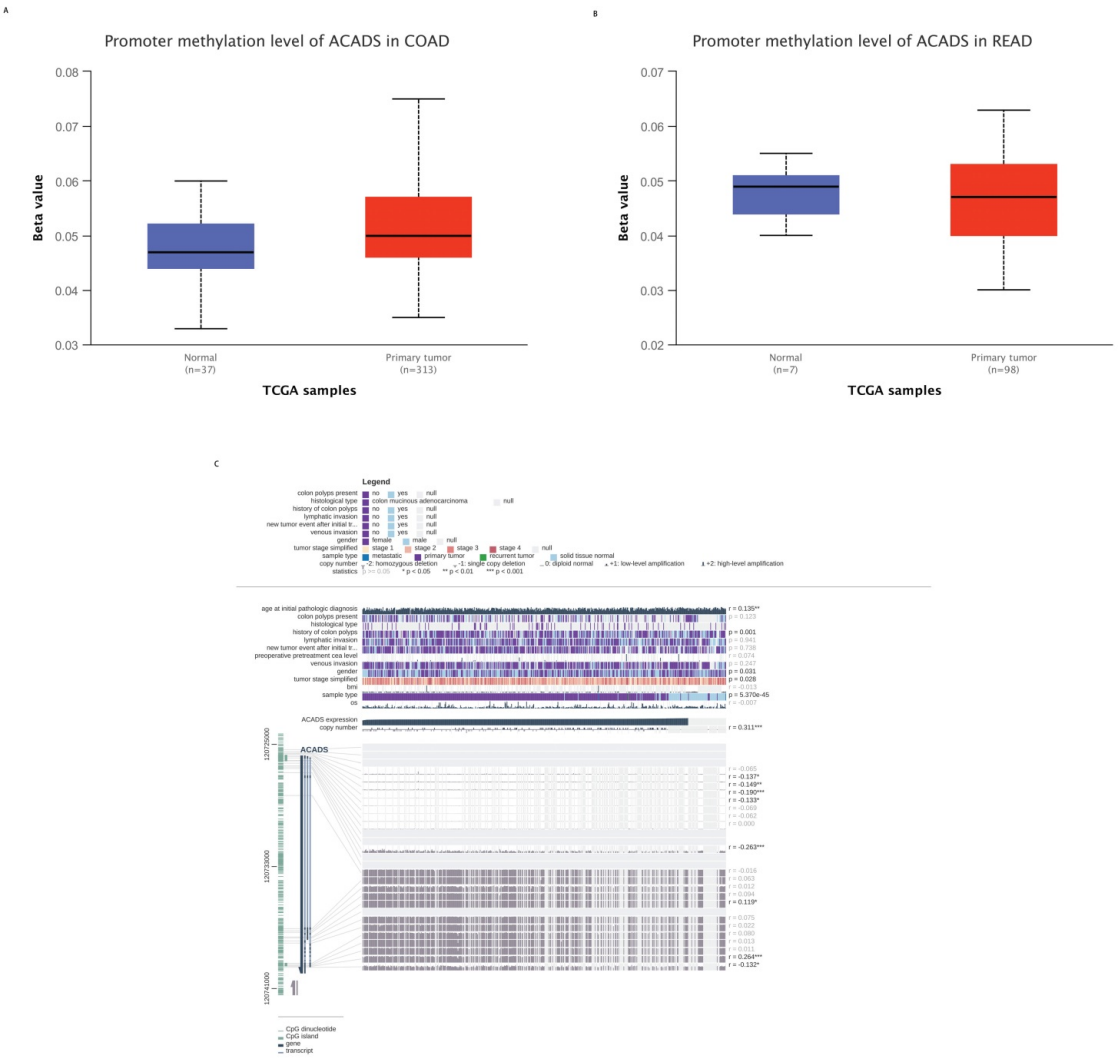
** Methylation levels of ACADS. (A)** Methylation levels of ACADS between colon adenocarcinoma and normal tissues. **(B)** Methylation levels of ACADS between rectum adenocarcinoma and normal tissues. **(C)** Methylation cites of ACADS in colon adenocarcinoma.

To explore the mechanisms of ACADS in CRC patients, we calculated correlations between its expression levels and methylation status utilizing public databases. First, we analyzed the difference in methylation levels between tumor tissues and normal tissues using the UALCAN database. The UALCAN analysis demonstrated that the methylation level of ACADS in normal tissues was significantly higher than that in tumor tissues. We further analyzed the methylation sites of ACADS using the MEXPRESS database. As shown in Figure 5, six methylation sites, including cg10600917, cg27522780, cg08618068, cg15631966, cg06793505, and cg00293381, in the DNA sequences of ACADS were negatively correlated with its expression levels, while cg01225086 and cg04618812 were positively correlated with its expression levels (Figure 6).

### Co-expression analysis of ACADS

**Figure 7:**
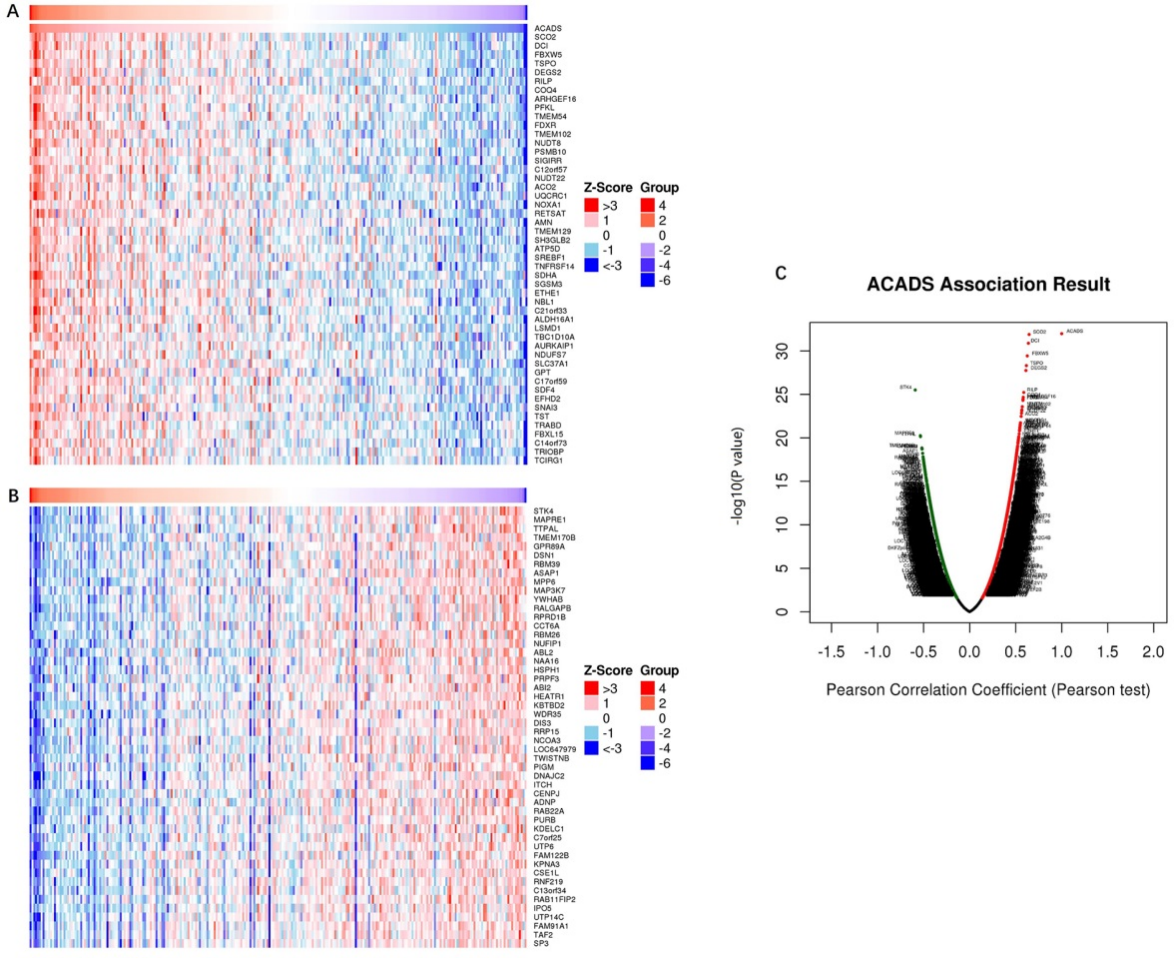
** Co-expression genes analysis. (A and B)** The heat map showing top 50 genes positively and negatively correlated with ACADS in colorectal cancer (CRC). **(C)** Correlations between ACADS and genes differentially expressed in CRC.

**Figure 8:**
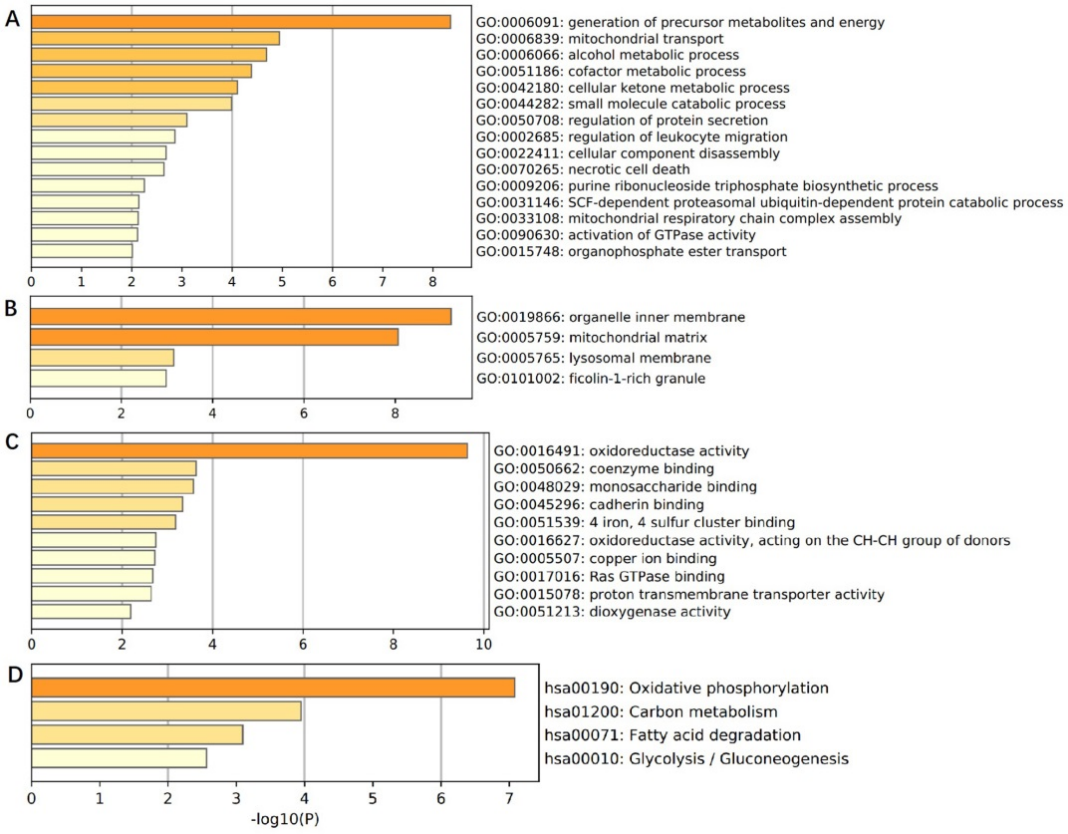
** Enrichment analysis of ACADS co-expressed genes. (A)** Biological processes. **(B)** Cellular components. **(C)** Molecular functions. **(D)** KEGG pathway.

In order to comprehensively understand the expression pattern of ACADS in CRC, the analysis of co-expression genes was performed by LinkedOmics (Figure 7). To analyze the common biological characteristics of co-expressed genes, we used Metascape to perform Gene Ontology (GO) and Kyoto Encyclopedia of Genes and Genomes (KEGG) analysis. The significant enriched biological processes included the generation of precursor metabolites and energy, mitochondrial process, and metabolic alcohol process (Figure 8A). In addition, inner organelle membrane, mitochondrial matrix, and lysosomal membrane were significantly enriched in cellular components (Figure 8B). For MF, the most significant entries were the activity of oxidoreductase, the activity of coenzyme binding, and monosaccharide binding (Figure 8C). Furthermore, the KEGG pathway analysis suggested that co-expressed genes were mainly enriched in oxidative phosphorylation, carbon metabolism, fatty acid degradation, and glycolysis/gluconeogenesis (Figure 8D). To sum up, these data indicated a vital role of ACADS in the regulation of cell mitochondrial metabolism.

### PPI network construction

**Figure 9:**
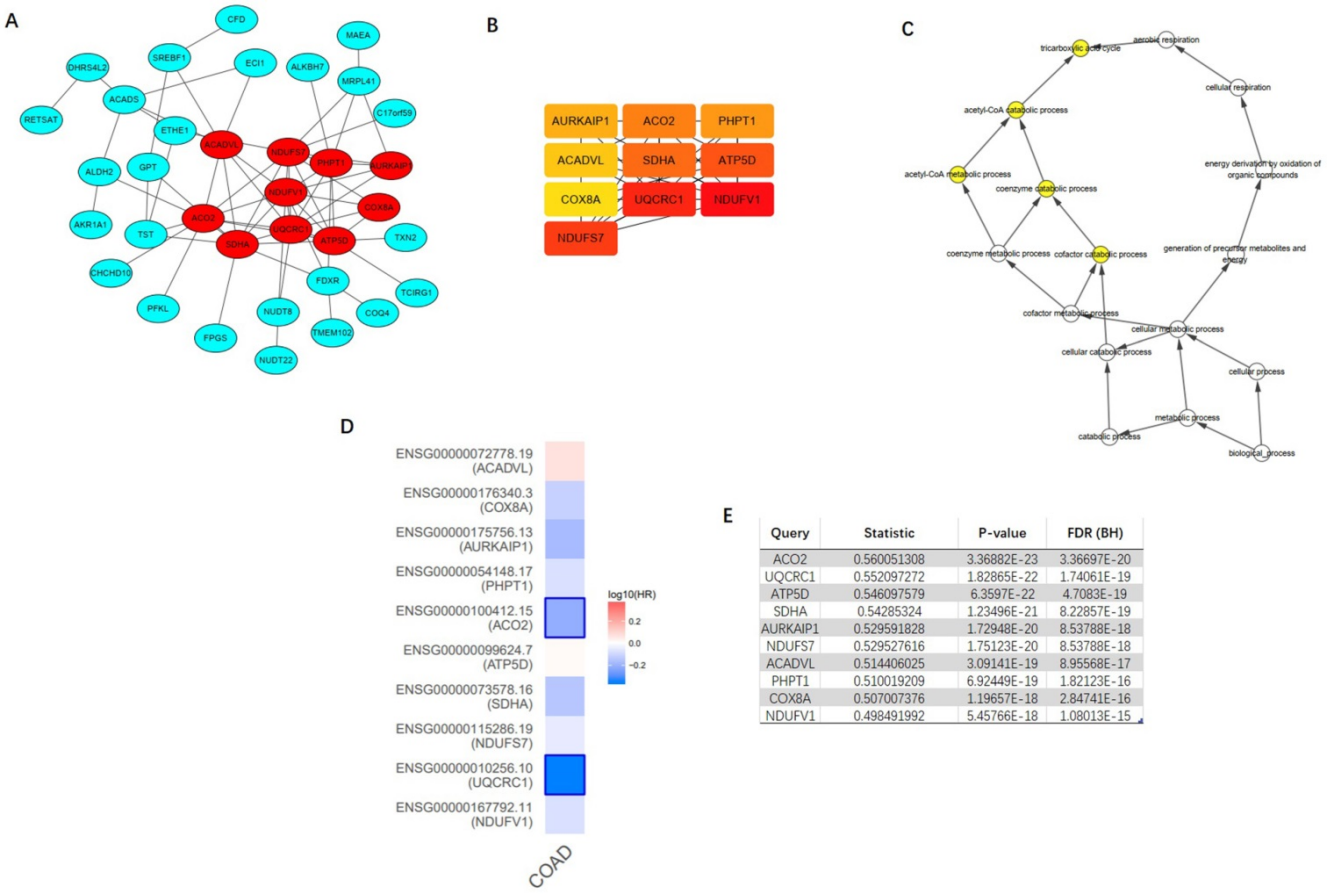
** Construction of protein-protein interaction (PPI) network of top 100 ACADS correlated genes and identification of hub genes. (A)** PPI network constructed by STRING. **(B)** Hub genes were selected via CytoHubba and MCODE plug-in in Cytoscape. **(C)** The biological process analysis of hub genes. **(D)** The survival map of hub genes. **(E)** The co-expression values of hub genes.

Top 100 positively correlated co-expressed genes were selected to construct the PPI network utilizing the STRING database. MCODE, a plug-in of the Cytoscape software, was applied to achieve the most important module (Figure 9A). CytoHubba was utilized to identify top ten hub genes, including NDUFV1, UQCRC1, NDUFS7, SDHA, ATP5D, ACO2, PHPT1, AURKAIP1, COX8A, and ACADVL (Figure 9B). The further GOBP analysis performed via BINGO plug-in indicated the hub genes mainly focused on the tricarboxylic acid cycle, acetyl-CoA catabolic process, acetyl-CoA metabolic process, coenzyme catabolic process and cofactor catabolic process, suggesting that they may participate in metabolism process (Figure 9C). Among top ten hub genes, ACO2 had the highest co-expression value and was selected out for further research (Figure 9E). A survival map based on TCGA of 10 hub genes was obtained from GEPIA (Figure 9D), which indicated that ACO2 might be a prognostic factor in CRC.

### Expression and prognostic values of ACO2

**Figure 10:**
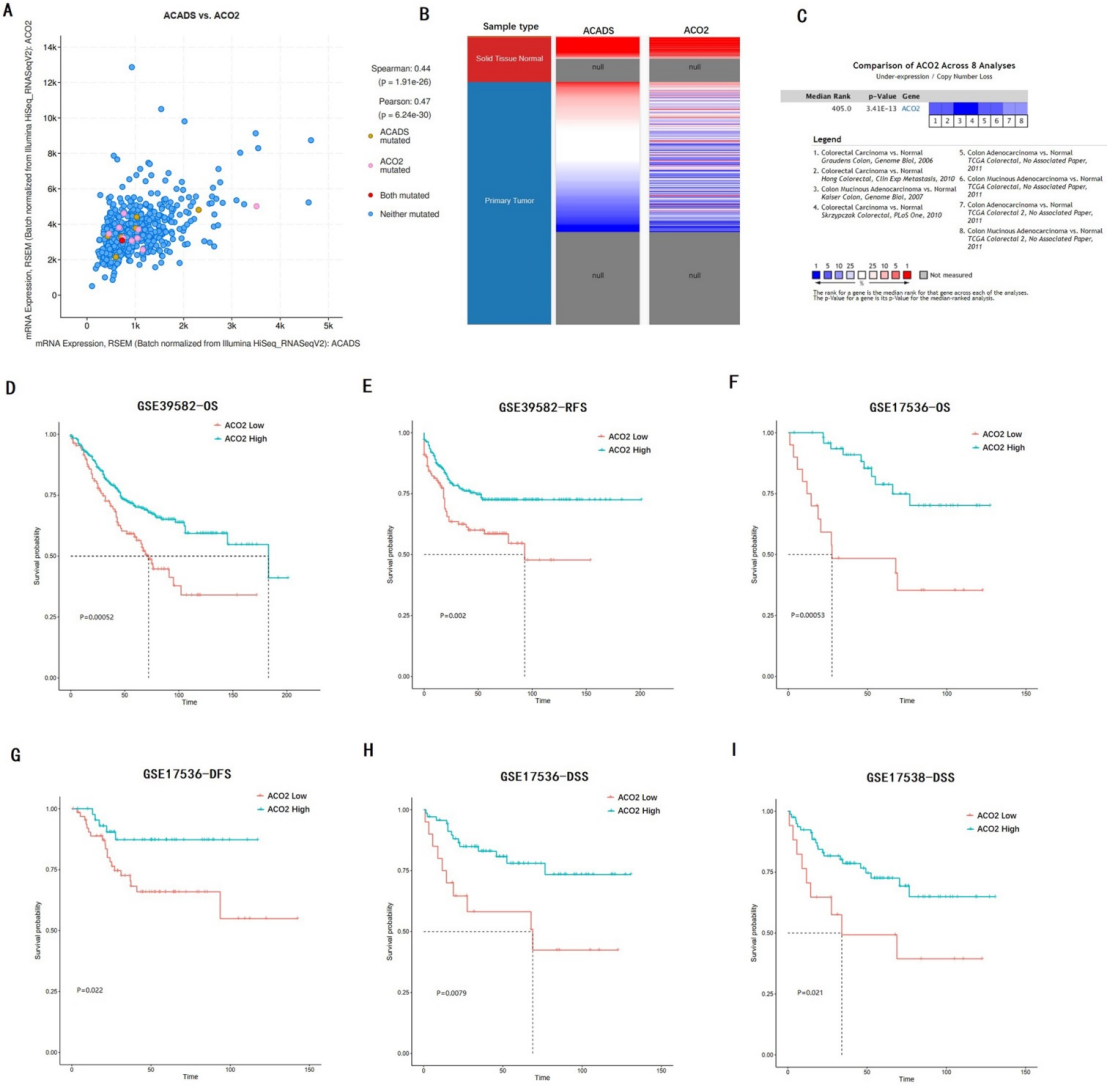
** Analysis of hub gene ACO2. (A)** The correlation between ACADS and ACO2. **(B)** Expression heatmap of ACADS and ACO2. **(C)** A meta-analysis of ACADS expression in 8 colorectal cancer studies. **(D-I)** Survival analysis of ACO2 based on the GEO database. OS, overall survival; RFS, relapse-free survival; DFS, disease-free survival; DSS, disease-specific survival.

We verified the co-expression between ACADS and ACO2 using cBioportal, and positive correlation coefficients were shown in Figure 10A. The correlation was further confirmed via hierarchical clustering in UCSC Cancer Genomics Browser (Figure 10B). Expression meta-analysis in 8 studies was obtained using the Oncomine database, and ACO2 expression was generally down-regulated in tumor tissue compared with normal tissue (Figure 10C). In addition, the prognostic value of ACO2 was confirmed by survival analysis in GSE39582, GSE17536, and GSE17538. It proved that low-level expression of ACO2 mRNA was closely related to the decreased OS, DFS, RFS, and disease-specific survival (DSS) in CRC patients (Figure 10D-I).

## Discussion

In the present study, results from multiple large databases demonstrated that ACADS expression was decreased in CRC tumor tissue in both mRNA and protein levels. The expression of ACADS was associated with clinical cancer stages and the ratio of CMS based on the analysis of TCGA and GEO database. In addition, several common immune checkpoint genes and MMR genes exhibited a significant relationship with ACADS expression levels. Further survival analysis indicated that low expression of ACADS could be a risk factor for CRC survival. Immune infiltration and methylation analysis suggested the potential of ACADS in CRC patients. Moreover, GO and KEGG enrichment analysis indicated that co-expressed genes of ACADS are expected mainly involved in cell mitochondrial metabolism. Interestingly, the selected hub gene ACO2, which had a strong positive correlation with ACADS, was closely associated with the survival of CRC patients.

Metabolic alteration is a common feature in the development of cancers 25, 26, and fatty acid metabolism plays a key role in several types of cancers, including colorectal cancer 27-29. As one of the key enzymes related to the metabolic process involved in tumorigenesis, it is greatly likely that ACADS might be a new diagnosis and treatment target. Studies showed that ACADS played a key role in some types of malignancies. ACADS was indicated as a potential methylation biomarker in hepatocellular carcinomas and was associated with cancer cell proliferation and metastasis 30. Adiponectin could repress the proliferation of breast cancer cells *in vitro*, and decreased the expression level of ACADS meanwhile, which indicated that ACADS could be a promising biomarker in breast cancer 31. As a part of a novel multi-dimensional transcriptome signature, ACADS participated in the prognosis prediction in bladder cancer 32. Previous studies showed that ACADS was altered in expression and related to the fatty acid metabolism pathway in CRC, which was then identified as a target of quercetin 33. However, the sample sizes of previous studies were small and conflicting results seemingly existed. Pira G et al. found that ACADS was down-regulated in tumor tissue in transcriptome analysis while Yeh CS et al. noted that ACADS was up-regulated in tumor cells stimulated by linoleic acid 11, 34. Our results confirmed that ACADS was decreased in CRC tumor tissue compared with paired normal tissue via multiple databases and systematic bioinformatic tools. In addition, ACADS was correlated with survival of CRC patients and could be a prognostic marker.

About 15-20% of CRC patients would be identified as a deficiency in mismatch repair (dMMR), which is demonstrated caused by mutations of MMR genes (MSH6, MSH2, MLH1, and PMS2) that code defective MMR proteins 35. Compared with mismatch-repair-proficient (pMMR) CRC patients, dMMR patients were shown to have better survival 36 and could benefit more from part of chemotherapy, target therapy, or immune therapy 37-39. Our current analysis showed that ACADS was correlated significantly with MMR gene PMS2 and immune checkpoint gene TNFRSF14, LGALS9, and TMIGD2. Low expression of ACADS largely predicted the unfavorable prognosis and was associated with higher proportion of pMMR patients, which indicated that low expression of ACADS may not good for the therapeutic effect and resulted in poorer survival.

Recently, it is widely recognized that mitochondria metabolism played a vital role in the development of cancers, including cancer growth, progression, and drug resistance 40. In addition, the changes in mitochondria metabolism can lead to tumor immune-suppression 41, which has an impact on the therapeutic effect of immunotherapy and the prognosis of cancer patients. In CRC, markers of mitochondrial metabolism indicated the poor outcome 42, and mitochondrial target drugs could inhibit the proliferation of colorectal cancer cells 43. Our results also showed that ACADS co-expressed genes mainly focused on cell mitochondrial metabolism, illustrating they might be promising target genes in the treatment of CRC. The current results showed that ACO2 had the most significant correlation with ACADS among ten hub genes. ACO2, the second enzyme involved in the tricarboxylic acid cycle (TCA), catalyzes citrate to isocitrate in mitochondria, which is vital in lipid metabolism 44, 45. Overexpression of ACO2 could reduce proliferation and weaken Warburg-like bioenergetic features of tumor cells in breast cancer. And ACO2 low expression was proven to be related to poor prognosis in gastric cancer 46. However, the expression of ACO2 is positively correlated with malignancy in prostate cancer 47. ACO2 was previously proved to be often deleted in CRC 48, and further mechanism research is warranted. We found that ACO2 was down-regulated in CRC and could be a protective factor for survival. Based on the present data, we speculated that co-targeting ACADS and ACO2, which function in mitochondria, might be an efficient way to fight against CRC.

Tumor microenvironment is believed to play a crucial role in tumorigenesis and cell proliferation 49. Tumor infiltrating lymphocytes (TILs) have been found in diverse cancer types, which can predict the prognosis of patients 50. Abnormally infiltrated immune cells in tumors promote tumor growth and escape from the host 51. Expression levels of ACADS in CRC patients were positively correlated with CD8+ T cells, which are activated by interacting with antigens presented through molecules on antigen presenting cells, playing an important role in inducing the anti-tumor immune response 52. Increasing the expression level of ACADS will not only help improve the prognosis of CRC patients, but may also lead to enhanced anti-tumor immune responses in CRC patients. CD4^+^ T cells are crucial in the development and activation of CD8^+^ T cells, also in the cellular immune response 53. The result of EPIC analysis demonstrated that ACADS expression levels were positively related to CD4^+^ T cells, which might promote CD8^+^ T cells activation and induce anti-tumor immune responses. Tumor-associated macrophages directly affect tumor progression. M1 macrophages play an anti-tumor effect, while M2 macrophages play a role in promoting tumor development 54. Our results indicated that ACADS expression levels were positively correlated with M1 macrophages, while negatively related to M2 macrophages. Targeting ACADS may further exert the anti-tumor effect of M1 macrophages, while inhibiting the tumor-promoting effect of M2 macrophages.

DNA methylation is a form of DNA chemical modification that can change genetic performance, playing a crucial role in regulating gene expression and the pathological process of tumor 55. We first calculated correlations between ACADS expression levels and methylation status using UALCAN. The results suggested that the methylation level of ACADS in normal tissues was significantly higher than that in tumor tissues, indicating that it is possible to influence the methylation of ACADS to change its gene expression to inhibit the development of CRC and improve the prognosis of CRC patients. Furthermore, we also predicted some possible methylation sites to help develop potential novel treatment options.

Nevertheless, there are some limitations in this study. First, this study was based on public databases, and lacked the verification of our own clinical data. The conclusion will be more convincing if we can use the data of patients recruited by our hospital. Further prospective studies should be carried out to explore the expression levels of ACADS in patients with CRC. Second, although qRT-PCR was performed to verify the expression level of ACADS, we did not explore its deeper mechanism of ACADS in patients with CRC, which is crucial for the development of new treatment strategies. Further mechanistic studies need to be conducted to investigate roles of ACADS in patients with CRC and to discover potential therapeutic targets.

## Conclusion

In the present study, diverse public databases were used to analyze and explore expression and roles of ACADS in CRC patients, providing multilevel evidences for the function of ACADS in prognostic prediction, immune infiltration, and methylation. ACADS might have the potential as the novel biomarker and therapeutic target in CRC patients.

## Supplementary Material

Supplementary figures and tables.

## Figures and Tables

**Table 1:** The sequence of primers

Gene		sequence
ACADS	Forward Primer	CGGCAGTTACACACCATCTAC
	Reverse Primer	GCAATGGGAAACAACTCCTTCTC
GAPDH	Forward Primer	GGAGCGAGATCCCTCCAAAAT
	Reverse Primer	GGCTGTTGTCATACTTCTCATGG
