# Supplementary material for: Integrated Analysis of Expression and Prognostic Values of Acyl-CoA Dehydrogenase short-chain in Colorectal Cancer
Source: Int J Med Sci. 2021 Sep 7;18(16):3631–43. doi: 10.7150/ijms.63953 (PMC8579304; doi:10.7150/ijms.63953)
Supplement: Supplementary file 1 — Supplementary figures and tables. [file ijmsv18p3631s1.pdf]

Table S1 The relationship between ACADS expression level and clinicopathological features (TCGA)

Variables	Total (n = 221)	ACADS Low (n = 114)	ACADS High (n = 107)	P value
**Age, Median (IQR)**	67 (56, 75)	66.5 (56.25, 76)	67 (55, 74.5)	0.636
**Gender, n (%)**				0.404
Male	121 (54.75)	66 (57.89)	55 (51.4)	
Female	100 (45.25)	48 (42.11)	52 (48.6)	
**BMI, Median (IQR)**	27.12 (23.93, 31.54)	27.58 (24.63, 33.51)	26.83 (23.43, 29.69)	0.11
**T.stage, n (%)**				0.082
Tis	5 (2.26)	1 (0.88)	4 (3.74)	
T1	37 (16.74)	15 (13.16)	22 (20.56)	
T2	146 (66.06)	78 (68.42)	68 (63.55)	
T3	31 (14.03)	20 (17.54)	11 (10.28)	
T4	2 (0.9)	0 (0)	2 (1.87)	
**N.stage, n (%)**				0.006
N0	131 (59.28)	56 (49.12)	75 (70.09)	
N1	56 (25.34)	35 (30.7)	21 (19.63)	
N2	34 (15.38)	23 (20.18)	11 (10.28)	
**M.stage, n (%)**				0.004
M0	152 (85.88)	71 (78.02)	81 (94.19)	
M1	25 (14.12)	20 (21.98)	5 (5.81)	
**Pathologic.stage, n (%)**				0.001
Stage I	40 (18.43)	15 (13.51)	25 (23.58)	
Stage II	85 (39.17)	36 (32.43)	49 (46.23)	
Stage III	67 (30.88)	40 (36.04)	27 (25.47)	
Stage IV	25 (11.52)	20 (18.02)	5 (4.72)	

**NLR, n (%)**				0.021
0≤LNR **<** 0.05	133 (65.84)	64 (60.38)	69 (71.88)	
0.05≤LNR **<** 0.19	32 (15.84)	18 (16.98)	14 (14.58)	
0.19≤LNR<0.39	15 (7.43)	6 (5.66)	9 (9.38)	
0.39≤LNR≤1.00	22 (10.89)	18 (16.98)	4 (4.17)	
**MSI, n (%)**				0.006
MSI-H	39 (18.4)	11 (10.09)	28 (27.18)	
MSI-L	37 (17.45)	21 (19.27)	16 (15.53)	
MSS	136 (64.15)	77 (70.64)	59 (57.28)	
**CEA, Median (IQR)**	3.1 (1.9, 7.05)	3.8 (1.85, 10.1)	2.8 (1.9, 5.5)	0.224
**CMS, n (%)**				< 0.001
CMS1	29 (17.06)	8 (9.3)	21 (25)	
CMS2	60 (35.29)	38 (44.19)	22 (26.19)	
CMS3	30 (17.65)	3 (3.49)	27 (32.14)	
CMS4	51 (30)	37 (43.02)	14 (16.67)	

Table S2 The relationship between ACADS expression level and clinicopathological features (GSE39582)

Variables	Total (n = 447)	ACADS Low (n = 93)	ACADS High(n = 354)	P value
**SEX, n (%)**				0.295
Male	245 (54.8098)	46 (49.4624)	199 (56.2147)	
Female	202 (45.1902)	47 (50.5376)	155 (43.7853)	
**LOCATION, n (%)**				0.066
Distal	262 (58.8764)	63 (67.7419)	199 (56.5341)	
Proximal	183 (41.1236)	30 (32.2581)	153 (43.4659)	

**TNM, n (%)**				0.002
Stage I	31 (6.9663)	4 (4.3011)	27 (7.6705)	
Stage II	209 (46.9663)	34 (36.5591)	175 (49.7159)	
Stage III	158 (35.5056)	36 (38.7097)	122 (34.6591)	
Stage IV	43 (9.6629)	19 (20.4301)	24 (6.8182)	
**T, n (%)**			ACADS High(n = 354)	0.971
Tis	3 (0.7026)	0 (0)	3 (0.8876)	
T1	8 (1.8735)	1 (1.1236)	7 (2.071)	
T2	40 (9.3677)	9 (10.1124)	31 (9.1716)	
T3	287 (67.2131)	59 (66.2921)	228 (67.4556)	
T4	88 (20.6089)	20 (22.4719)	68 (20.1183)	
**N, n (%)**				0.093
N0	242 (57.2104)	41 (46.0674)	201 (60.1796)	
N1	102 (24.1135)	27 (30.3371)	75 (22.4551)	
N2	75 (17.7305)	20 (22.4719)	55 (16.4671)	
N3	4 (0.9456)	1 (1.1236)	3 (0.8982)	
**M, n (%)**				< 0.001
M0	382 (89.8824)	70 (78.6517)	312 (92.8571)	
M1	43 (10.1176)	19 (21.3483)	24 (7.1429)	
**CHEMOTHERAPY.ADJUVANT, n (%)**				0.272
Yes	173 (39.7701)	40 (45.4545)	133 (38.3285)	
No	262 (60.2299)	48 (54.5455)	214 (61.6715)	
**KRAS, n (%)**				0.311
Wild type	247 (59.9515)	58 (65.1685)	189 (58.5139)	
Mutation	165 (40.0485)	31 (34.8315)	134 (41.4861)	
**BRAF, n (%)**				0.657

Wild type	344 (89.1192)	72 (91.1392)	272 (88.5993)	
Mutation	42 (10.8808)	7 (8.8608)	35 (11.4007)	
**MMR, n (%)**				0.015
dMMP	59 (14.4608)	5 (5.7471)	54 (16.8224)	
pMMP	349 (85.5392)	82 (94.2529)	267 (83.1776)	
**TP53, n (%)**				0.003
Wild type	136 (49.8168)	21 (32.8125)	115 (55.0239)	
Mutation	137 (50.1832)	43 (67.1875)	94 (44.9761)	
**CMS, n (%)**				< 0.001
CMS1	74 (19.0722)	9 (10.3448)	65 (21.5947)	
CMS2	169 (43.5567)	42 (48.2759)	127 (42.1927)	
CMS3	59 (15.2062)	6 (6.8966)	53 (17.608)	
CMS4	86 (22.1649)	30 (34.4828)	56 (18.6047)	

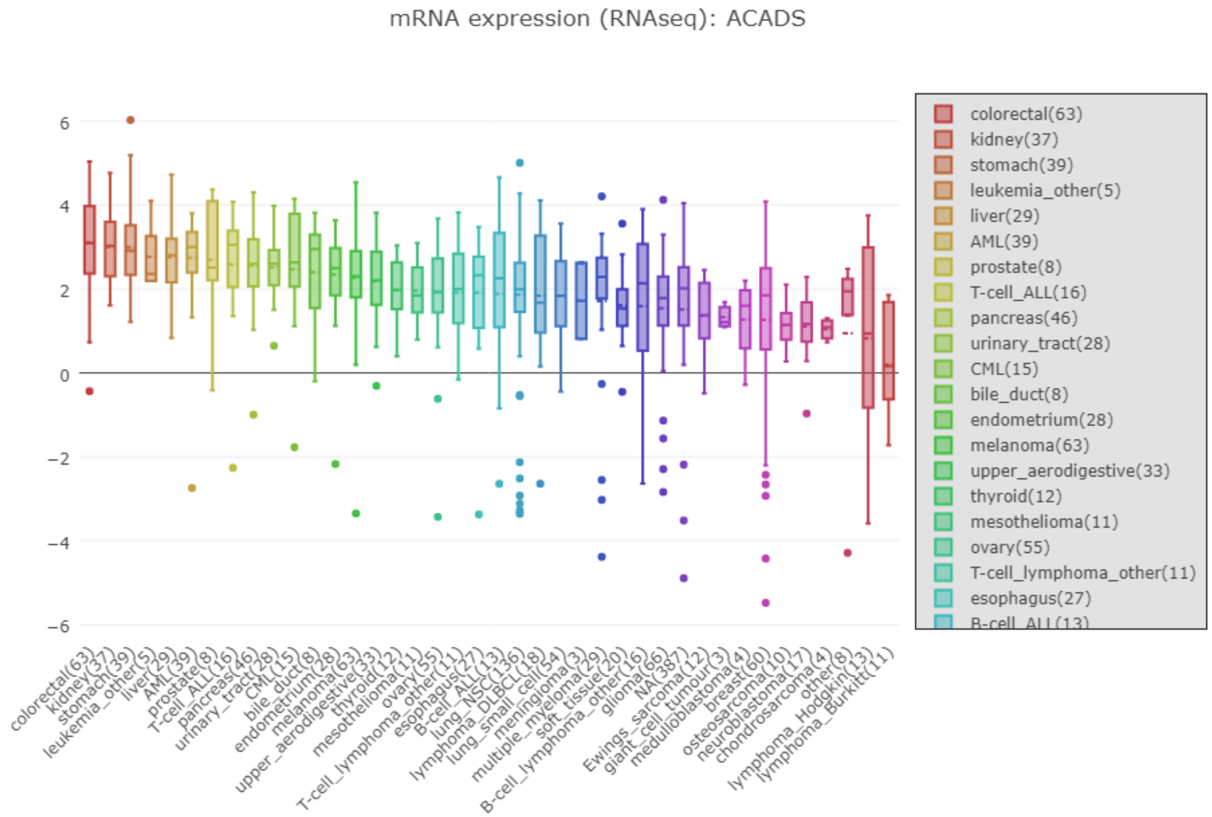

Figure S1 The mRNA expression levels of ACADS in the CCLE database.

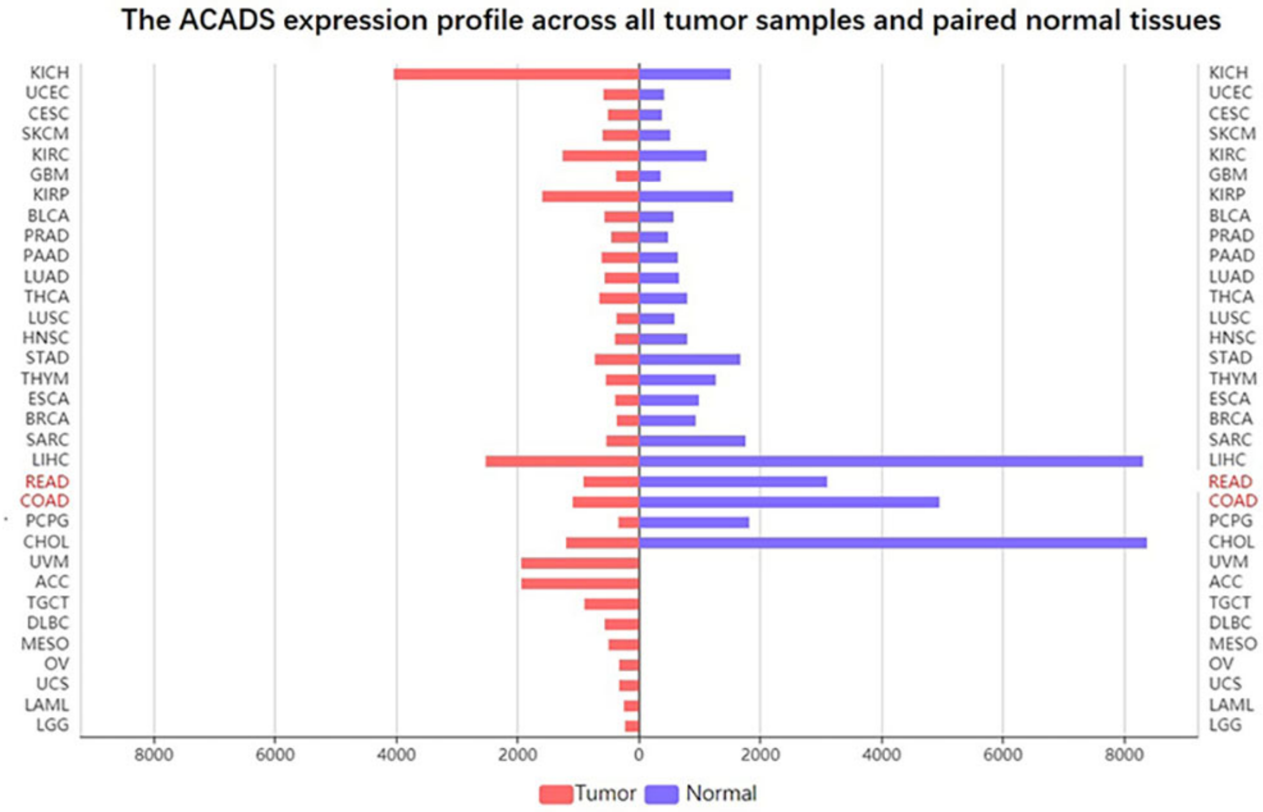

Figure S2 The ACADS expression profile across all tumor samples and paired normal tissues in TCGA.

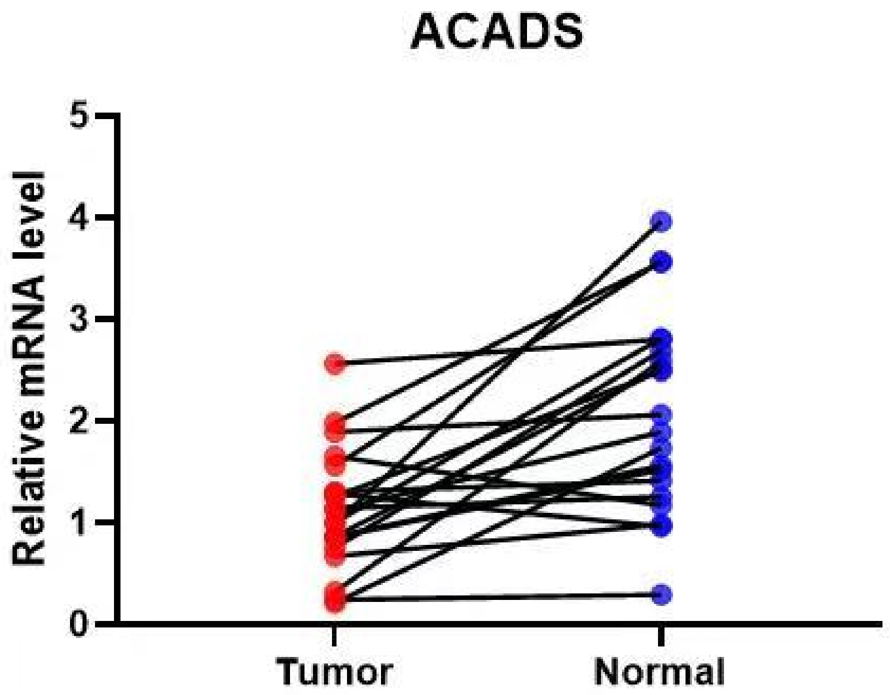

Figure S3 The relative expression of ACADS between tumor and normal tissues.

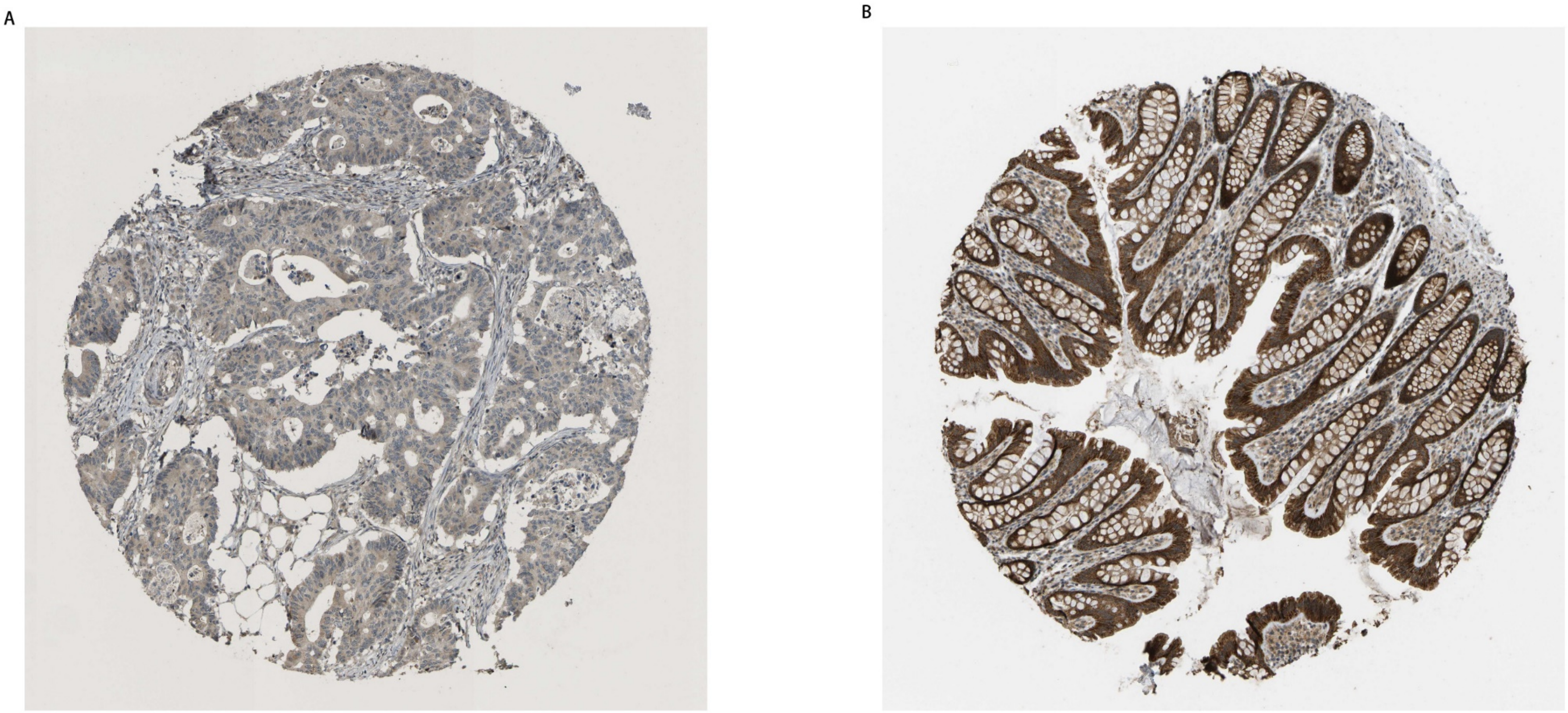

Figure S4 The protein levels of ACADS using immunohistochemistry. **(A)** Tumor tissues with low staining levels. **(B)** Normal tissues with high staining levels.

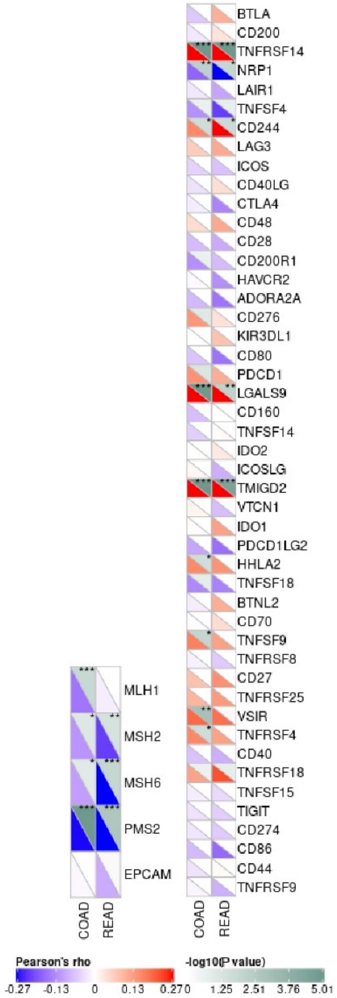

Figure S5 The ACADS co-expression signature of common immune checkpoint genes and mismatch repair (MMR) genes.
